# Do vulnerable groups access prevention services? Cervical cancer screening and HIV testing among homeless migrant women in the Paris metropolitan area

**DOI:** 10.1371/journal.pone.0255900

**Published:** 2021-08-13

**Authors:** Lorraine Poncet, Henri Panjo, Virginie Ringa, Armelle Andro

**Affiliations:** 1 UVSQ, Univ. Paris-Sud, Inserm, Primary Care and Prevention Team, CESP, Université Paris-Saclay, Villejuif, France; 2 French Collaborative Institute on Migration, Paris, France; 3 Institute of Demography, Université Paris I Pantheon-Sorbonne, Paris, France; Katholieke Universiteit Leuven Rega Institute for Medical Research, BELGIUM

## Abstract

**Introduction:**

Homeless migrant women, facing adverse living conditions and barriers to legal status, are at risk of cervical cancer, HIV infection and may encounter barriers to screening services. We investigate factors associated with each screening in a population of migrant women in France and aim to determine the mean time since last HIV testing according to duration of residence in France.

**Methods:**

We use data from the DSAFHIR study (Rights and Health of Migrant Women in Emergency Housing) investigating health and migration experience of homeless migrant women housed in emergency housing hotels in the Paris Metropolitan area in 2017. We computed multivariate logistic regression models to investigate no lifetime cervical cancer screening (CCS) and no lifetime HIV test. We used linear regression models to analyze time since last HIV test.

**Results:**

We included 469 women. 46% of respondents had no lifetime CCS, 31% had no lifetime HIV test. Both screenings were associated with educational attainment and French proficiency. Compared with duration of residence < 1 year, duration ≥ 7 years was associated with a lower likelihood of no lifetime CCS (adjusted Odd Ratio = 0.17; 95% CI = 0.07–0.39). Compared to women born in North Africa, women born in West (aOR = 0.15; 95% CI = 0.07–0.33) and East Africa (aOR = 0.06; 95% CI = 0.02–0.20) were less likely to have no lifetime HIV test. Time since last HIV test increased for each additional year spent in France (coef = 0.21; 95% CI = 0.09, 0.33).

**Conclusion:**

While access to CCS remains poor for recent migrants, HIV testing is more likely to occur shortly after migration.

## Introduction

Cervical cancer is the ninth most common cancer affecting women in the WHO European region, with 67,000 new cases and 31,000 deaths in 2020 [1]. In France, around 3000 new cases and 1100 deaths are reported each year [2]. Large disparities exist between regions in Europe, with higher incidence rates in Central and Eastern Europe compared with Northern and Southern Europe [1]. New HIV diagnoses among women totaled 50,000 cases in the WHO European Region in 2018 [3], with 1700 occurring in France in 2018 [4]. Large disparities exist within the region, with greater incidence and mortality rates in Eastern Europe [3].

In Europe, migrant women from low-income countries are at higher risk of developing cervical cancer than non-migrant women, due to higher prevalence of human papillomavirus (HPV) in their country of origin and insufficient access to screening [5, 6]. They are also more likely to be diagnosed at an advanced stage of the disease, leading to worse health outcomes than the host population [6]. In France cervical cancer screening (CCS) is carried out by Pap smear. At the time of the study, it was recommended every three years for women aged 25 to 65 years, after two initial tests one year apart. HPV tests were introduced in 2019, to be carried out every five years for women above 30 years old. Self-sampling HPV testing was recommended in the French guidelines in 2019 [7] but were not available at the time of the study. Pap smears are carried out by gynecologists, general practitioners, and midwives. The test is reimbursed under the National Health Insurance (NHI), with no upfront payment for low-income patients [2]. Migrant women with legal residency status are eligible to the NHI. Undocumented migrant women are eligible to a specific health coverage plan for undocumented people, also covering CCS at no cost for patients. CCS coverage was 59% in France in 2015–2017 [8], with large regional and social disparities. Previous studies have found that low educational level, low income and being unemployed were risk factors for being overdue for screening [9, 10]. Migrant women in the Paris area were more likely to have delayed and no lifetime CCS compared to women of French origin [11].

In the EU, it was estimated that approximately 40% of new HIV cases were reported among migrants [4]. Migrant women in France constitute the group with the second highest incidence rate for new HIV infections [12, 13]. In 2017, 75% of new infections through heterosexual transmission concerned immigrants, primarily from Sub Saharan Africa [12]. As in other European countries [14], the high infection rate in this group is due to high HIV prevalence in countries of origin, but also due to HIV infections in France: it was estimated that 30% of HIV-positive migrant women from Sub Saharan Africa are infected after migration [15]. Migrant men and women are also more likely to be diagnosed late, negatively impacting treatment, mortality [16] and virus transmission [12, 17]. With economic deprivation increasing infection risk [18], it is crucial that migrant women be tested repeatedly in the years following migration. They are indeed specifically targeted for screening: while the French National Authority for Health recommended at least one lifetime HIV screening for the general population, it recommended yearly HIV testing among migrants from countries with high HIV prevalence, specifically Sub Saharan Africa and the Caribbean [13]. HIV testing is available in France as blood tests and rapid tests in various medical and non-medical settings, reimbursed under the NHI, and available for free in screening centers and non-governmental organizations (NGOs).

While much is known concerning both HIV testing and cervical cancer screening among migrant populations, we focus here on a group of migrant women who encounter several layers of deprivation, living in housing instability, financial stress, and uncertainty regarding administrative status. The health care system in France, with universal health care coverage, is meant to be accessible to all. In the case of these two screenings, the public health objective is to reach out to and include the most disadvantaged groups [13, 19]. Analyzing both screenings together helps to gauge the capacity of universal health care systems to be inclusive of the most vulnerable groups in our societies. With different access and target populations, the two screenings illustrate and nuance access to primary prevention programs for marginalized groups.

Our objective was to identify factors associated with CCS and HIV testing in a population of vulnerable migrant women. We further aimed to determine the mean time since last HIV testing in this group, according to duration of residence in France.

## Methods

### Data

We used data from the DSAFHIR study (Rights and Health of Housed Migrant and Refugee Women), carried out in 2017.

### Population

The study investigated the health needs and obstacles in accessing healthcare services of homeless migrant women housed in low-end hotels as an emergency housing option in the Paris Metropolitan area in France (N = 469). Women aged 25 to 40 are over-represented in this population, as housing women with children is a priority of the emergency housing system. Migrant families face obstacles accessing resources and service providers from hotels situated in distant suburban areas. Moreover, as immigration policies have restricted access to long-term residence permits, access to public healthcare coverage is hampered, further challenging access to care.

### Data collection and study setting

A sampling based on a two-stage non-random selection was used to select geographical zones and hotels. All migrant women aged 18 years or more housed in the selected hotels were eligible. The questionnaire was administered in the respondents’ hotel rooms, in April and May 2017. Respondents were offered to participate in sexual health interventions and a follow-up survey eight months after inclusion to evaluate the interventions. We used here only data collected at inclusion. The design of the study was detailed elsewhere [20].

### Data tool

The questionnaire was administered face to face in ten different languages (French, Russian, Romanian, Arabic, Bambara, Soninke, Georgian, Armenian), by multilingual female surveyors. As much as possible, questionnaire sections of existing surveys were used. Prior to data collection, the questionnaire was pilot-tested, and it was reviewed by a committee of women with lived experience of emergency housing in hotels.

### Ethics

We paid particular attention to the quality of informed consent. The multilingual surveyors made sure respondents understood the goal of the study and the possibility to refuse to participate. In particular, we insisted that participating or refusing to participate would not have any impact—positive or negative—on their housing situation.

Ethical approval for the DSAFHIR study was granted by the People Protection Committee for medical research on March 30, 2017 (CPP reference number: IDRCB number 2016-A02005-46).

### Variables of interest

#### CCS

**Fig 1:**
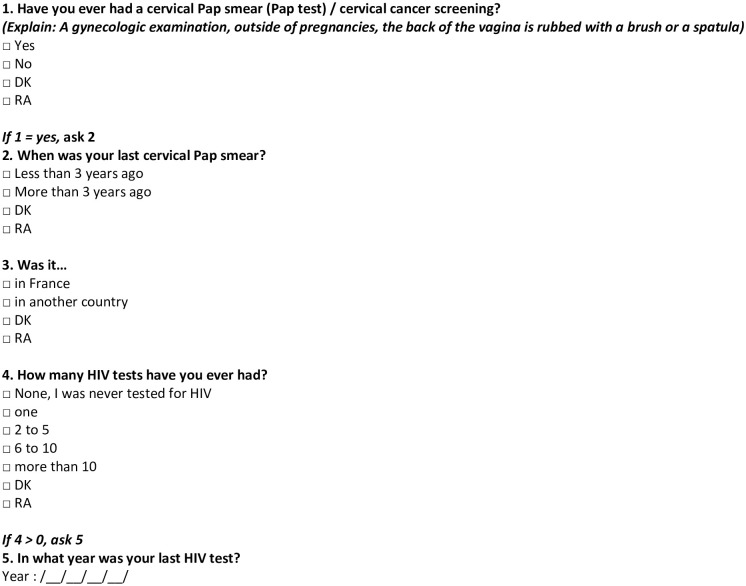
DSAFHIR study questionnaire—Variables of interest.

Respondents were asked whether they ever had a Pap smear, and time (more or less than 3 years prior) and country of last test (see Fig 1). Our outcome was ‘No lifetime CCS’.

#### HIV

Respondents were asked their lifetime number of HIV tests and year of the last test. Our outcome was ‘No lifetime HIV test’. The year of last HIV test was used to compute time since last HIV test.

### Independent variables

We tested the association between each screening and variables informing on socio-demographic characteristics, access to healthcare services and general health. Socio-demographic characteristics included age, educational level, duration of residence in France, relationship status, self-assessed knowledge of French language and paid work in the last 12 months (long or short-term, declared or not). We investigated access to care through medical insurance coverage, visit to a general practitioner (GP) and to a gynecologist in the last 12 months, having given birth in France and owning a public transportation card. General health was investigated through a measure of self-perceived physical health. Current depression was assessed using the Mini International Neuropsychiatric Interview (French version 5.0.0, 1998) [21].

### Statistical analyses

We ran bivariate analyses to identify factors associated with each of the two screenings using Chi2 tests. Variables with p-value < 0.2 were selected for multivariate analysis in logistic regression models. Variables were then backward-selected manually at the 0.2 threshold. Knowledge of French language was removed from multivariate analyses because of correlation with region of origin and educational level.

We ran the same analyses fitting maximum-likelihood two-equations probit models (bivariate probit) with simultaneous estimation of equations associated with the two outcomes of interest [22]. Bivariate probit model is a generalization of the probit model used to estimate two correlated binary outcomes jointly. The method adjusts for the potential residual correlation of our two outcomes of interest, and yields in this case unbiased estimates. Because the p-value of the residual correlation coefficient rho was greater than 0.05 (p = 0.19), therefore not confirming the correlation of error terms after controlling for the independent variables, and because our results showed no difference from the results estimated with simple logistic regression models, we chose to present the multivariate logistic regression models for each outcome (see S1 Table for bivariate probit model).

We computed mean number of years since last HIV test according to duration of residence. P-value was computed using a simple linear regression predicting time since last HIV test based on duration of residence as a categorical variable.

A simple linear regression was calculated to predict time since last HIV test based on duration of residence as a continuous variable. Covariates (age, educational level, relationship status, access to health professionals) were chosen according to hypotheses suggested by the literature of variables associated with both HIV screening and duration of residence and likely to confound their association and were then added into a multivariate linear regression model.

Because of different no lifetime HIV test rates between women from Sub Saharan Africa and women from other regions, and because HIV testing recommendations differ for the two groups [13], we analyzed the timing of HIV testing according to duration of residence separately for women born in Sub Saharan Africa and women from other regions.

### Analytical sub-samples

Concerning CCS, we included respondents aged 25 to 65 years, corresponding to the age range for CCS in the French screening guidelines. We excluded 10 respondents with non-valid value for CCS realization (missing = 1; ‘I don’t know’ = 9). Analytical sample for CCS included 387 respondents.

HIV testing analyses included respondents of all ages. We excluded respondents with missing values (n = 2) or answering ‘I don’t know’ (n = 37) for number of lifetime HIV tests. Analytical sample for HIV test included 430 respondents.

For analyses of time since last HIV test, analyses were carried out on 268 respondents with a valid date of last HIV test.

All analyses were performed using Stata 15.

## Results

### Population description

One third of respondents were aged below 30 years and 46% were aged between 30 and 40 years (Table 1). One third of the respondents had no schooling or only primary education, and 18% pursued higher education. 56% of the respondents were born in Sub Saharan Africa. 41% of the sample were undocumented, and 21% were uninsured. Two thirds (70%) of respondents had not worked in the past year. French language proficiency was unevenly distributed, with 41% of respondents with a very good command of French and 20% with very poor proficiency. Two thirds of the sample had given birth in France. 33% of respondents had no GP visit in the past year, and 57% had no gynecologist visit. 56% of the respondents reported good self-perceived health status, while 45% had current depression.

**Table 1:** Respondents’ characteristics, DSAFHIR study.

	N	%
	469	100
Age (yrs)		
< 30	153	32.6
[30–40]	215	45.8
[40–50]	72	15.4
50 +	28	6.0
Education		
No schooling	62	13.2
Primary	61	13.0
Secondary	143	30.5
High school diploma	107	22.8
Higher education	83	17.7
Relationship status		
In a couple	276	58.9
Not in a couple	193	41.1
Region of origin		
North Africa/Middle East	65	13.9
West Africa	166	35.4
East/Central Africa	97	20.7
Former Soviet/Yugoslavian States	57	12.2
European Union	56	11.9
Others	28	6.0
French language proficiency		
Very good	192	40.9
Good	54	11.5
Average	76	16.2
Poor	53	11.3
Very poor	94	20.0
Duration of residence (yrs)		
<1	84	17.9
[1–2]	61	13.0
[2–3]	62	13.2
[3–4]	55	11.7
[4–5]	40	8.5
[5–6]	39	8.3
[6–7]	31	6.6
> = 7	97	20.7
Medical insurance coverage		
National health insurance	181	38.8
Health insurance for undocumented	185	39.7
No coverage	100	21.3
GP^1^ visit in last 12 months		
No	155	33.0
Yes	314	67.0
Gynecologist visit in last 12 months		
No	266	56.7
Yes	203	43.3
Having given birth in France		
No	157	33.5
Yes	312	66.5
Own public transportation card		
No	309	65.9
Yes	160	34.1
Paid work in last 12 months		
No	330	70.4
Yes	139	29.6
Administrative status		
Long-term	138	29.4
Short-term	97	20.7
Asylum seeker	40	8.5
Undocumented	190	40.5
Met with friend in last month		
No	151	32.2
Yes	318	67.8
Self-rated health		
Very good/good	263	56.1
Other	206	43.9
Current depression		
No	259	55.2
Yes	210	44.8

^1^GP: general practitioner

### CCS

#### Screening participation rates

46% of respondents had no lifetime CCS (Table 2). The great majority of Pap smears took place in France, with only 4% of respondents having a last Pap smear abroad (not shown in tables).

**Table 2:** Characteristics associated with no lifetime cervical cancer screening, bivariate and multivariate analyses, DSAFHIR study.

	N	%	p^1^	OR	95% CI	aOR^2^ N = 376	95% CI
	176/387	45.5					
Age (yrs)							
< 30	41/82	50.0	0.14	1.00		1.00	
[30–40]	99/211	46.9		0.88	0.53–1.47	1.24	0.68–2.28
[40–50]	23/69	33.3		0.50	0.26–0.97	0.86	0.38–1.92
50 +	13/25	52.0		1.08	0.44–2.65	3.18	0.98–10.3
Education							
No schooling	37/54	68.5	0.001	2.37	1.20–4.69	1.94	0.87–4.33
Primary	22/50	44.0		0.86	0.44–1,67	0.74	0.35–1.57
Secondary	55/115	47.8		1.00		1.00	
High school diploma	36/84	42.9		0.82	0.46–1.44	0.84	0.44–1.60
Higher education	22/74	29.7		0.46	0.25–0.86	0.46	0.23–0.93
Relationship status							
In a couple	116/222	52.3	0.002	1.00		1.00	
Not in a couple	60/165	36.4		0.52	0.35–0.79	0.46	0.26–0.81
Region of origin							
North Africa/Middle East	27/58	46.6	0.001	1.00		1.00	
West Africa	66/136	48.5		1.08	0.58–2.00	1.24	0.57–2.68
East/Central Africa	27/85	31.8		0.53	0.27–1.06	0.78	0.33–1.83
Former Soviet/Yugoslavian States	17/50	34.0		0.59	0.27–1.29	0.48	0.20–1.17
European Union	26/37	70.3		2.71	1.13–6.50	2.62	0.94–7.27
Others	13/21	61.9		1.87	0.67–5.18	2.02	0.62–6.55
French language proficiency							
Very good	57/158	36.1	0.003				
Good	26/48	54.2					
Average	24/62	38.7					
Poor	25/46	54.4					
Very poor	44/73	60.3					
Duration of residence (yrs)							
<1	42/62	67.7	0.001	1.00		1.00	
[1–2]	24/47	51.1		0.50	0.23–1.09	0.55	0.22–1.35
[2–3]	29/56	51.8		0.51	0.24–1.08	0.54	0.23–1.24
[3–4]	14/48	29.2		0.20	0.09–0.44	0.21	0.09–0.53
[4–5]	12/30	40.0		0.32	0.13–0.78	0.32	0.11–0.89
[5–6]	14/34	41.2		0.33	0.14–0.79	0.31	0.11–0.82
[6–7]	14/28	50.0		0.48	0.19–1.19	0.46	0.16–1.28
> = 7	27/82	32.9		0.23	0.12–0.47	0.17	0.07–0.39
Medical insurance coverage							
National health insurance	56/147	38.1	0.05				
Health insurance for undocumented	78/162	48.2					
No coverage	41/76	54.0					
GP visit in last 12 months							
No	69/127	54.3	0.01	1.00		1.00	
Yes	107/259	41.3		0.59	0.39–0.91	0.65	0.39–1.08
Gynecologist visit in last 12 months							
No	118/218	54.1	<0.0001	1.00		1.00	
Yes	58/168	34.5		0.45	0.30–0.68	0.49	0.30–0.80
Having given birth in France							
No	72/135	53.3	0.02				
Yes	104/252	41.3					
Own public transportation card							
No	120/249	48.2	0.15				
Yes	56/138	40.6					
Paid work in last 12 months							
No	131/272	48.2	0.10				
Yes	45/115	39.1					
Administrative status							
Long-term	50/107	46.7	0.74				
Short-term	35/87	40.2					
Asylum seeker	14/31	45.2					
Undocumented	75/159	47.2					
Met with friend in last month							
No	60/128	46.9	0.72				
Yes	116/258	45.0					
Self-rated health							
Very good/good	99/211	46.9	0.53				
Other	77/176	43.8					
Current depression							
No	75/169	44.4	0.81				
Yes	75/174	43.1					

^1^Chi^2^ tests;

^2^Analyses adjusted simultaneously on age, educational attainment, relationship status, region of origin, duration of residence, GP visit in last 12 months, gynecologist visit in last 12 months

#### Factors associated with no lifetime CCS

In bivariate analyses, no lifetime CCS was significantly associated with socio-demographic characteristics such as educational level, French language proficiency, relationship status and region of origin. Two thirds (69%) of respondents with no schooling had no lifetime CCS, versus only one third (30%) of respondents with higher education (p = 0.001). 60% of respondents with a very poor proficiency in French had no lifetime CCS, versus only 36% of those with very good command of French (p = 0.003). Women in a relationship were more often never screeners (52% vs 36%, p = 0.002). Respondents with longer length of residence had significantly lower rates of no lifetime screening (p < 0.0001). Respondents with no health coverage were more often never screeners (54%) than respondent with national health coverage (38%, p = 0.05). Access to healthcare professionals was associated with no lifetime screening: 41% of women who had visited a GP in the last year and 35% of women who had visited a gynecologist were never screened, while 54% of women with no GP (p = 0.01) or gynecologist (p < 0.0001) were never screened. 41% of women who had given birth in France had not been screened, while this was the case for 53% of women who had not given birth in France (p = 0.02).

In multivariate analyses, educational level, relationship status, duration of residence and no visit to a gynecologist remained associated with no lifetime CCS (Table 2). Compared to women with secondary education, women with higher education were less likely to have no lifetime CCS (adjusted Odd Ratio = 0.46; 95% CI = 0.23–0.93). Single or separated women were less likely not to have been screened (aOR = 0.46; 95% CI = 0.26–0,81). Women with duration of residence of 3 years or more were less likely to have never been screened, compared with recently arrived women. Women who had visited a gynecologist in the past 12 months were also less likely not to have been screened (aOR = 0.49; 95% CI = 0.30–0.80).

### HIV

#### Screening participation rates

31% of respondents had no lifetime HIV test (Table 3).

**Table 3:** Characteristics associated with no lifetime HIV test, bivariate and multivariate analyses, DSAFHIR study.

	N	%	p^1^	OR	95% CI	aOR^2^ N = 414	95% CI
	135/430	31.4					
Age (yrs)							
< 30	53/142	37.3	0.03	1.00		1.00	
[30–40]	47/193	24.4		0.54	0.34–0.87	0.70	0.39–1.24
[40–50]	25/69	36.2		0.95	0.53–1.73	1.22	0.58–2.57
50 +	10/25	40.0		1.12	0.47–2.67	0.72	0.22–2.36
Education							
No schooling	22/54	40.7	0.02	1.27	0.66–2.44	1.72	0.78–3.81
Primary	20/54	37.0		1.09	0.56–2.10	1.38	0.63–3.01
Secondary	46/131	35.1		1.00		1.00	
High school diploma	25/102	24.5		0.60	0.34–1.07	0.66	0.33–1.31
Higher education	15/77	19.5		0.45	0.23–0.87	0.43	0.20–0.95
Relationship status							
In a couple	104/244	42.6	<0.0001	1.00		1.00	
Not in a couple	31/185	16.8		0.27	0.17–0.43	0.57	0.32–1.03
Region of origin							
North Africa/Middle East	29/53	54.7	<0.0001	1.00		1.00	
West Africa	34/158	21.5		0.23	0.12–0.44	0.15	0.07–0.33
East/Central Africa	5/93	5.4		0.05	0.02–0.13	0.06	0.02–0.20
Former Soviet/Yugoslavian States	24/49	49.0		0.79	0.36–1.73	0.91	0.40–2.07
European Union	32/51	62.8		1.47	0.67–3.25	0.85	0.36–2.02
Others	11/26	42.3		0.61	0.24–1.57	0.71	0.26–1.96
French language proficiency							
Very good	34/185	18.4	<0.0001				
Good	15/47	31.9					
Average	27/68	39.7					
Poor	24/49	49.0					
Very poor	35/81	43.2					
Duration of residence (yrs)							
<1	27/78	34.6	0.39				
[1–2]	13/52	25.0					
[2–3]	15/60	25.0					
[3–4]	15/51	29.4					
[4–5]	10/39	25.6					
[5–6]	11/37	29.7					
[6–7]	9/29	31.0					
> = 7	35/84	41.7					
Medical insurance coverage							
National health insurance	49/166	29.5	0.71				
Health insurance for undocumented	53/169	31.4					
No coverage	32/93	34.4					
GP visit in last 12 months							
No	49/143	34.3	0.37				
Yes	86/286	30.1					
Gynecologist visit in last 12 months							
No	85/248	34.3	0.14				
Yes	50/181	27.6					
Having given birth in France							
No	59/149	39.6	0.008				
Yes	76/281	27.1					
Own public transportation card							
No	100/281	35.6	0.008	1.84	1.17–2.89	1.72	1.00–2.98
Yes	34/147	23.1		1.00		1.00	
Paid work in last 12 months							
No	92/297	31.0	0.78				
Yes	43/133	32.3					
Administrative status							
Long-term	48/123	39.0	0.21				
Short-term	25/91	27.5					
Asylum seeker	11/39	28.2					
Undocumented	51/174	29.3					
Met with friend in last month							
No	42/138	30.4	0.75				
Yes	93/291	32.0					
Self-rated health							
Very good/good	69/245	28.2	0.09				
Other	66/185	35.7					
Current depression							
No	51/183	27.8	0.19				
Yes	67/196	34.2					

^1^Chi^2^ tests;

^2^Analyses adjusted simultaneously on age, educational attainment, relationship status, region of origin, owning public transportation card

#### Factors associated with no lifetime HIV test

In bivariate analyses, women with no schooling were never tested for HIV twice as often as women with higher education (41% vs 20%, p = 0.02). Only 18% of women with very good command of French language had never been tested for HIV, compared to 49% of women with poor language proficiency and 43% of women with very poor proficiency (p<0.0001). Women in a relationship had never been tested more often than single women (43% vs 17%, p<0.0001). Women from Sub Saharan Africa had the lowest rates of no lifetime HIV test. Owning a public transportation pass was associated with a lower rate of no HIV testing (23% vs 36%, p = 0.008). 27% of women who had given birth in France had not been tested, while this was the case for 40% of women who had had not given birth in France.

In multivariate analyses, educational level, relationship status, region of origin and owning a public transportation card were associated with no lifetime HIV testing (Table 3). Compared to women with secondary education, women with higher education were less likely not to have been tested (aOR = 0.43; 95% CI = 0.20–0.95), but there was no difference for women with no schooling. Compared to women from North Africa and the Middle East, women from West Africa and East or Central Africa were less likely not to have been tested (aOR = 0.15; 95% CI = 0.07–0.33 and aOR = 0.06; 95% CI = 0.02–0.20). Women with no public transportation card were more likely not to have been tested (aOR = 1.72; 95% CI = 1.00–2.98).

### Time since last HIV test

The mean number of years since last HIV test was 1.85 years in the total sample and increased from 1.35 years in newly-arrived migrant women to 2.56 years in women who had been in France for 7 years or more (p = 0.04) (Table 4). A similar trend was observed among women from Sub Saharan Africa, with mean number of years since last HIV test increasing from 1.47 years in women in France for less than one year to 2.76 years in women with the longest duration of residence (p = 0.007). In simple linear regression, we found that time since last HIV test increased 0.20 for each additional year spent in France (95% CI = 0.07, 0.32, p = 0.002). A similar coefficient was found when adjusting for age, educational level, relationship status and recent visit to GP and gynecologist. Stratifying analyses on region of origin, similar results were found for women born in Sub Saharan Africa in bivariate (coef = 0.20; 95% CI = 0.04, 0.36; p = 0.01) and multivariate analyses (coef = 0.19; 0.04, 0.34; p = 0.01). Among women born in other regions, time since last HIV test increased 0.18 for each additional year spent in France (95% CI = 0.01, 0.34, p = 0.002) in bivariate analyses, and 0.27 in multivariate analyses (95% CI = 0.07, 0.47, p = 0.01).

**Table 4:** Mean time since last HIV test (in years), bivariate and multivariate linear regression, by region of origin, DSAFHIR study.

	All			Sub Saharan			Non-Sub Saharan		
	n = 268			n = 200			n = 68		
	Mean (yrs)	95% CI	p	Mean (yrs)	95% CI	p	Mean (yrs)	95% CI	p
Total	1.85	1.60, 2.11		1.78	1.49, 2.08		2.07	1.59, 2.56	
Duration of residence (yrs)			0.04			0.007			0.25
<1	1.35	0.84, 1.85		1.47	0.81, 2.13		1.07	0.24, 1.89	
[1–2]	1.25	0.79, 1.71		0.92	0.56, 1.28		2.00	0.69, 3.31	
[2–3]	1.74	1.27, 2.21		1.64	1.19, 2.08		2.33	-0.29, 4.96	
[3–4]	1.59	1.18, 2.01		1.61	1.14, 2.07		1.50	-0.09, 3.09	
[4–5]	2.07	1.51, 2.63		2.00	1.43, 2.57		2.50	-0.81, 5.81	
[5–6]	2.58	1.23, 3.94		2.21	0.22, 4.20		3.10	1.00, 5.19	
[6–7]	2.31	1.33, 3.30		1.50	0.05, 2.95		2.80	1.38, 4.22	
> = 7	2.56	1.56, 3.57		2.76	1.51, 4.00		1.75	1.01, 2.49	
Test of linearity			0.002			0.01			0.03
	Coef	95% CI	p	Coef	95% CI	p	Coef	95% CI	p
Duration of residence (yrs)	0.20	0.07, 0.32	0.002	0.20	0.04, 0.36	0.01	0.18	0.01, 0.34	0.03
Duration of residence (yrs)^1^	0.21	0.09, 0.33	0.001	0.19	0.04, 0.34	0.01	0.27	0.07, 0.47	0.01

^1^Multivariate model adjusted on age, educational attainment and relationship status, visit to GP in last 12 months, visit to gynecologist in last 12 months

## Discussion

We found that 46% of the respondents had never been screened for cervical cancer, and 31% had never received an HIV test. Women with higher educational attainment, with better French proficiency and women who were not in a relationship were more likely to have received the screenings. Women with a long duration of residence and women who had visited a gynecologist in the last year were more likely to have received a Pap smear, while women from Sub Saharan Africa were more likely to have been tested for HIV.

Because data is scarce concerning this specific population, it is difficult to make international comparisons. However, studies from across Europe have consistently reported low CCS participation among migrant women, and their results show that the most vulnerable migrant women are the most likely not to be screened: migrant women with low socio-economic status [23–27], low social support [26, 28], and with short duration of residence [23, 25, 28]. Concerning HIV testing, a study carried out in 2015 in several European countries [14] has found that access to HIV testing for migrant women was better for women born in Africa, for women with permanent residency and for women who had received prenatal care in the host country.

Analyses from the SIRS (French acronym for health, inequalities and social ruptures) cohort, a representative sample of adult French-speaking population of the Paris Metropolitan area, found that 9% of the total eligible population had no lifetime CCS [10], and that 26% of immigrant women had no lifetime CCS [11] in 2010. Because the cohort data were collected only in French, women encountering obstacles to CCS due to a poor command of French and generally poor knowledge of the healthcare system were excluded, accounting for the higher rate of no lifetime screening in our results. In a sample of homeless women housed in emergency shelters in the Paris metropolitan area in 2013, researchers found no lifetime CCS rates similar to our own [29].

In 2018, after this survey was conducted, an organized screening program for cervical cancer was initiated in France, where women with delayed screening are invited by post. While it is undoubtedly beneficial for increasing screening participation rates, it may not reach migrant women in unstable housing, whose affiliation to the national health insurance system is periodically jeopardized by precarious administrative status. Self-sampling has been shown to improve screening participation for under-screened women [30] and will soon be available in France [7]. Although self-sampling might prove a helpful option, it would not reach women with no health coverage and might be difficult to carry out for women with little command of written French. Outreach services, such as mobile screening clinics, might improve access to CCS. Additionally, as a large share of this population has given birth in France, increasing CCS in prenatal care might be an efficient way to reach homeless migrant women.

Concerning HIV testing, a study conducted in the general population in the Paris metropolitan area in 2010 found that 22% of women aged 18 to 54 had no lifetime HIV test [31]. Analyses from the SIRS cohort [32] found that 33% of women were never tested, and 41% among immigrant women. The better outcome in our population can be explained by the fact that women from Sub Saharan Africa represented 56% of our sample (versus 26% of immigrant women included in the SIRS cohort [33]) with a low rate of no-lifetime HIV test contributing to drive down the global rate of no lifetime HIV test in our sample and likely accounting for the disparity. The lower likelihood of no lifetime HIV test among women from Sub Saharan Africa has been noted before [33] and reflects the French HIV testing guidelines of repeated HIV testing in migrants from Sub Saharan Africa and the Caribbean. It can also reflect testing opportunities before migration, in countries with high HIV prevalence.

Women with higher education were more likely to have received both screenings, consistent with a large body of literature on the role of educational attainment on health [34, 35] and health promotion behaviors [36, 37]. This speaks to the increased health literacy, knowledge about infections and testing opportunities among people with higher education, and increased ability to request it or to be receptive when testing is offered. It could also result from differentiated practices of health professionals, possibly prompter to interact with more educated patients. A previous study carried out in the Paris region has found that even among women with a recent medical visit [10, 38], higher educational attainment was associated with higher CCS rates.

It should be noted that compared to women with secondary education, women with no schooling were not more likely to have no lifetime CCS or no lifetime HIV test. Previous studies investigating hepatitis B [39, 40] or HIV [32, 41] screening have found that people in precarious living conditions could be screened more often, resulting from public health and service providers’ policies to target vulnerable groups.

We found that owning a public transportation card, allowing people to access service providers, is associated with more HIV testing. This resonates with a public debate in the Paris metropolitan area where special public transportation discount for undocumented migrants was cancelled by conservative regional leadership in 2016 [42]. Our results illustrate the direct impact of such misguided policies on public health and the fight against HIV/AIDS in particular.

Women who had been in France for 3 years or more were more likely to have been screened for cervical cancer than women who migrated within the year. This is in line with generally better access to care gained with longer time in the host country, and more opportunities for repeated screening. However, duration of residence was not associated with lifetime HIV testing: greater familiarity with the health care system with longer duration of residence did not provide benefits for HIV testing. This suggests that HIV testing occurs mostly shortly after migration and before migration. Our results confirm that time since the last test increased with each additional year spent in France. This is consistent with previous research that has shown that the median first-time HIV test occurred during the second year in France among Sub Saharan migrants [41] and that time since last HIV test increased with number of years spent in France [17]. This is problematic because it was estimated that one third of HIV positive migrant women from Sub Saharan Africa were infected after migration [15], and therefore need to be tested regularly in the years following migration. Women in our sample are especially exposed to the risk of HIV infection in France because they have no personal housing and live in poverty [18]: they are more likely to have risky sexual encounters, to trade sex, and to have poor negotiating power for safe sex.

Our results suggest that CCS realization demands a long-term integration in the health care system while HIV testing is restricted to recently arrived migrants, as if the risk of infection existed only upon arrival. A long duration of residence in the host country might act as a signal to health professionals that HIV test is superfluous. Long-term integration in the health care system seems beneficial for CCS, but detrimental to HIV testing.

### Limitations and strengths

As respondents lived in the Paris metropolitan area, our results can not be extended to migrant women living in other regions of France, where screening possibilities may differ. Because other CCS method exist in some respondents’ countries of origin, such as visual inspection with acetic acid, asking only about Pap smear realization may restrict our measure of lifetime cervical cancer screening occurring before migration. As data was self-reported, recall and reporting biases may exist. To help with comprehension, surveyors systematically explained what the Pap smear is and how it is carried out.

The survey was conducted in several different languages, in a population who usually remains unheard and out of reach of large public surveys. There was no medical setting bias, and we could reach women with very poor or no access to health services.

## Conclusion

We found poor access to cervical cancer screening for recently arrived migrant women and social inequalities in accessing both screenings. Our results argue for early offer of CCS and for repeated and sustained testing for HIV. Better awareness of migrant women’s poor living conditions may help health professionals understand the heightened risk of infection and offer relevant screening.

## Supporting information

S1 Table Characteristics associated with one or both screenings, biprobit multivariate regression, DSAFHIR study. (DOCX)

S2 Table Characteristics associated with no lifetime cervical cancer screening and no lifetime HIV test, bivariate and multivariate logistic regression analyses, DSAFHIR study. (DOCX)

S3 Table Mean time since last HIV test (in years), multivariate linear regression, by region of origin, DSAFHIR study. (DOCX)

## References

[1] Ferlay J, Ervik M, Lam F, Colombet M, Mery L, Pineros M, et al. Global Cancer Observatory: Cancer Today. Lyon, France: International Agency for Research on Cancer; 2020. https://gco.iarc.fr/today

[2] Institut National du Cancer. Dépistage du cancer du col de l’utérus. 2019. https://www.e-cancer.fr/Acces-thematique/Depistage-du-cancer-du-col-de-l-uterus

[3] Mårdh O, Quinten C, Kuchukhidze G, Seguy N, Dara M, Amato-Gauci AJ, et al. HIV among women in the WHO European Region—epidemiological trends and predictors of late diagnosis, 2009–2018. Euro Surveill. 2019;24. doi: 10.2807/1560-7917.ES.2019.24.48.1900696 31796153 PMC6891943

[4] European Centre for Disease Prevention and Control, World Health Organization, Regional Office for Europe. HIV/AIDS surveillance in Europe 2019–2018 data. Stockholm; 2019.

[5] WHO. Report on the health of refugees and migrants in the WHO European Region. No public health without refugee and migrant health. 2019.

[6] Di Felice E, Caroli S, Paterlini L, Campari C, Prandi S, Giorgi Rossi P. Cervical cancer epidemiology in foreign women in Northern Italy: role of human papillomavirus prevalence in country of origin. Eur J Cancer Prev. 2015;24: 223–230. doi: 10.1097/CEJ.0000000000000137 25714783

[7] Haute Autorité de Santé. Evaluation de la recherche des papillomavirus humains (HPV) en dépistage primaire des lésions précancéreuses et cancéreuses du col de l’utérus et de la place du double immuno-marquage p16/Ki67. 2019. https://www.has-sante.fr/jcms/c_2806160/fr/evaluation-de-la-recherche-des-papillomavirus-humains-hpv-en-depistage-primaire-des-lesions-precancereuses-et-cancereuses-du-col-de-l-uterus-et-de-la-place-du-double-immuno-marquage-p16/ki67

[8] Hamers F, Jezeweski-Serra D. Couverture du dépistage du cancer du col de l’utérus en France, 2012–2017. Bulletin Epidemiologique Hebdomadaire. 2019; 417–23.

[9] Vallée J, Cadot E, Grillo F, Parizot I, Chauvin P. The combined effects of activity space and neighbourhood of residence on participation in preventive health-care activities: The case of cervical screening in the Paris metropolitan area (France). Health & Place. 2010;16: 838–852. doi: 10.1016/j.healthplace.2010.04.009 20451439

[10] Grillo F, Vallée J, Chauvin P. Inequalities in cervical cancer screening for women with or without a regular consulting in primary care for gynaecological health, in Paris, France. Prev Med. 2012;54: 259–265. doi: 10.1016/j.ypmed.2012.01.013 22296836

[11] Rondet C, Lapostolle A, Soler M, Grillo F, Parizot I, Chauvin P. Are immigrants and nationals born to immigrants at higher risk for delayed or no lifetime breast and cervical cancer screening? The results from a population-based survey in Paris metropolitan area in 2010. PLoS ONE. 2014;9: e87046. doi: 10.1371/journal.pone.0087046 24466323 PMC3899363

[12] Bulletin de Santé Publique. Découvertes de séropositivité VIH et diagnostics de SIDA—France 2018. 2019.

[13] Haute Autorité de Santé. Réévaluation de la stratégie de dépistage de l’infection à VIH en France. 2017.

[14] Fakoya I, Álvarez-del Arco D, Woode-Owusu M, Monge S, Rivero-Montesdeoca Y, Delpech V, et al. A systematic review of post-migration acquisition of HIV among migrants from countries with generalised HIV epidemics living in Europe: mplications for effectively managing HIV prevention programmes and policy. BMC Public Health. 2015;15: 561. doi: 10.1186/s12889-015-1852-9 26085030 PMC4472169

[15] Desgrées-du-Loû A, Pannetier J, Ravalihasy A, Gosselin A, Supervie V, Panjo H, et al. Sub-Saharan African migrants living with HIV acquired after migration, France, ANRS PARCOURS study, 2012 to 2013. Euro Surveill. 2015;20. doi: 10.2807/1560-7917.ES.2015.20.46.30065 26607135

[16] Montlahuc C, Guiguet M, Abgrall S, Daneluzzi V, de Salvador F, Launay O, et al. Impact of late presentation on the risk of death among HIV-infected people in France (2003–2009). J Acquir Immune Defic Syndr. 2013;64: 197–203. doi: 10.1097/QAI.0b013e31829cfbfa 24047970

[17] Lapostolle A, Massari V, Chauvin P. Time since the last HIV test and migration origin in the Paris metropolitan area, France. AIDS Care. 2011;23: 1117–1127. doi: 10.1080/09540121.2011.554522 21500026 PMC3472401

[18] Desgrees-du-Lou A, Pannetier J, Ravalihasy A, Le Guen M, Gosselin A, Panjo H, et al. Is hardship during migration a determinant of HIV infection? Results from the ANRS PARCOURS study of sub-Saharan African migrants in France. AIDS. 2016;30: 645–656. doi: 10.1097/QAD.0000000000000957 26558722 PMC4732006

[19] Ministère des Solidarités et de la Santé. Stratégie nationale de santé 2018–2022. 2017. https://solidarites-sante.gouv.fr/IMG/pdf/dossier_sns_2017_vdefpost-consult.pdf

[20] Poncet, Andro A, Eberhard M, Fleury M, Riou F, Gelly M, et al. Investigating and Improving Access to Reproductive Healthcare for Vulnerable Migrant Women in France. Social Science Protocols. 2019;2: 1–13. doi: 10.7565/ssp.2019.2672

[21] Sheehan DV, Lecrubier Y, Sheehan KH, Amorim P, Janavs J, Weiller E, et al. The Mini-International Neuropsychiatric Interview (M.I.N.I.): the development and validation of a structured diagnostic psychiatric interview for DSM-IV and ICD-10. J Clin Psychiatry. 1998;59 Suppl 20: 22–33;quiz 34–57. 9881538

[22] Cappellari L, Jenkins SP. Multivariate Probit Regression using Simulated Maximum Likelihood: The Stata Journal. 2003 [cited 23 Jul 2020]. doi: 10.1177/1536867X0300300305

[23] Hertzum-Larsen R, Kjær SK, Frederiksen K, Thomsen LT. Participation in cervical cancer screening among immigrants and Danish-born women in Denmark. Prev Med. 2019;123: 55–64. doi: 10.1016/j.ypmed.2019.02.023 30796926

[24] Otero L, Sanz B, Blasco T. Early detection of cervical cancer according to the discourses of primary care midwives in Segovia, Spain. Rev Saude Publica. 2011;45: 824–830. doi: 10.1590/s0034-89102011005000061 21845292

[25] Møen KA, Kumar B, Qureshi S, Diaz E. Differences in cervical cancer screening between immigrants and nonimmigrants in Norway: a primary healthcare register-based study. Eur J Cancer Prev. 2017;26: 521–527. doi: 10.1097/CEJ.0000000000000311 27749381 PMC5627531

[26] Brzoska P, Aksakal T, Yilmaz-Aslan Y. Utilization of cervical cancer screening among migrants and non-migrants in Germany: results from a large-scale population survey. BMC Public Health. 2020;20: 5. doi: 10.1186/s12889-019-8006-4 31906964 PMC6945536

[27] Marques P, Nunes M, Antunes M da L, Heleno B, Dias S. Factors associated with cervical cancer screening participation among migrant women in Europe: a scoping review. Int J Equity Health. 2020;19: 160. doi: 10.1186/s12939-020-01275-4 32917224 PMC7488650

[28] Barrera-Castillo M, Fernández-Peña R, Del Valle-Gómez MDO, Fernández-Feito A, Lana A. [Social integration and gynecologic cancer screening of immigrant women in Spain]. Gac Sanit. 2020;34: 468–473. doi: 10.1016/j.gaceta.2019.01.002 30929951

[29] Vuillermoz C, Vandentorren S, Roze M, Rondet C, Chauvin P. Cervical cancer screening among homeless women in the Greater Paris Area (France): results of the ENFAMS survey. Eur J Cancer Prev. 2017;26: 240–248. doi: 10.1097/CEJ.0000000000000225 26895575

[30] Reaching women who do not participate in the regular cervical cancer screening programme by offering self-sampling kits: A systematic review and meta-analysis of randomised trials. European Journal of Cancer. 2015;51: 2375–2385. doi: 10.1016/j.ejca.2015.07.006 26296294

[31] ORS Ile-de-France. Les connaissances, attitudes, croyances et comportements face au VIH/Sida en Ile de France en 2010. 2011.

[32] Massari V, Lapostolle A, Grupposo M-C, Dray-Spira R, Costagliola D, Chauvin P. Which adults in the Paris metropolitan area have never been tested for HIV? A 2010 multilevel, cross-sectional, population-based study. BMC Infect Dis. 2015;15: 278. doi: 10.1186/s12879-015-1006-9 26198690 PMC4509770

[33] Lapostolle A, Massari V, Beltzer N, Halfen S, Chauvin P. Differences in recourse to HIV testing according to migration origin in the Paris metropolitan area in 2010. J Immigr Minor Health. 2013;15: 842–845. doi: 10.1007/s10903-012-9742-z 23099525 PMC4901159

[34] Marmot, Ryff CD, Bumpass LL, Shipley M, Marks NF. Social inequalities in health: Next questions and converging evidence. Social Science & Medicine. 1997;44: 901–910. doi: 10.1016/s0277-9536(96)00194-3 9080570

[35] Lleras-Muney A. The Relationship Between Education and Adult Mortality in the United States. Rev Econ Stud. 2005;72: 189–221. doi: 10.1111/0034-6527.00329

[36] Chandola, Clarke P, Morris JN, Blane D. Pathways between education and health: a causal modelling approach. Journal of the Royal Statistical Society: Series A (Statistics in Society). 2006;169: 337–359. doi: 10.1111/j.1467-985X.2006.00411.x

[37] Régnier F, Masullo A. Obésité, goûts et consommation. Revue francaise de sociologie. 2009;Vol. 50: 747–773.

[38] Rigal L, Saurel-Cubizolles M-J, Falcoff H, Bouyer J, Ringa V. Do social inequalities in cervical cancer screening persist among patients who use primary care? The Paris Prevention in General Practice survey. Prev Med. 2011;53: 199–202. doi: 10.1016/j.ypmed.2011.06.016 21726576

[39] Dray-Spira R, Gigonzac V, Vignier N. Les immigrés subsahariens suivis pour une hépatite B chronique: caractéristiques et accès au diagnostic et aux soins. La Découverte. Parcours de vie et santé des Africains immigrés en France. La Découverte. La Découverte; 2017. pp. 195–206. https://www.cairn.info/parcours-de-vie-et-sante-des-africains-immigres—9782707196453-page-195.htm

[40] Rigal L, Rouessé C, Collignon A, Domingo A, Deniaud F. Facteurs liés à l’absence de proposition de dépistage du VIH-sida et des hépatites B et C aux immigrés en situation de précarité. Revue d’Épidémiologie et de Santé Publique. 2011;59: 213–221. doi: 10.1016/j.respe.2011.01.007 21724346

[41] Limousi F, Lert F, du Loû AD, Dray-Spira R, Lydié N, Group PS. Dynamic of HIV-testing after arrival in France for migrants from sub-Saharan Africa: The role of both health and social care systems. PLOS ONE. 2017;12: e0188751. doi: 10.1371/journal.pone.0188751 29267347 PMC5739385

[42] Act Up Paris. Ile-de-France Mobilités: Seulement si vous avez des papiers. Mediapart. 31 Jul 2018. https://blogs.mediapart.fr/act-paris/blog/310718/ile-de-france-mobilites-seulement-si-vous-avez-des-papiers-0. Accessed 25 Jul 2020.

