# Supplementary material for: Do vulnerable groups access prevention services? Cervical cancer screening and HIV testing among homeless migrant women in the Paris metropolitan area
Source: PLoS One. 2021 Aug 13;16(8):e0255900. doi: 10.1371/journal.pone.0255900 (PMC8363022; doi:10.1371/journal.pone.0255900)
Supplement: S1 Table — (DOCX) [file pone.0255900.s001.docx]

**S1 Table. Characteristics associated with one or both screenings, biprobit multivariate regression, DSAFHIR study, N = 346**

|  |  | No lifetime CCS | | | | No lifetime HIV test | | | |
| --- | --- | --- | --- | --- | --- | --- | --- | --- | --- |
|  |  | Coef | 95% CI | Standard error | p | Coef | 95% CI | Standard error | p |
|  | Age (yrs) |  |  |  |  |  |  |  |  |
|  | < 30 | Ref |  |  |  | Ref |  |  |  |
|  | [30-40[ | -0.02 | -0.38, 0.34 | 0.19 | 0.89 | -0.08 | -0.49, 0.33 | 0.21 | 0.69 |
|  | [40-50[ | -0.22 | -0.71, 0.26 | 0.25 | 0.36 | 0.20 | -0.31, 0.72 | 0.26 | 0.43 |
|  | 50 + | 0.53 | -0.18, 1.24 | 0.36 | 0.14 | -0.27 | -1.09, 0.55 | 0.42 | 0.51 |
| Education | |  |  |  |  |  |  |  |  |
|  | No schooling | 0.55 | 0.7, 1.04 | 0.25 | 0.03 | 0.34 | -0.16, 0.84 | 0.26 | 0.18 |
|  | Primary | -0.17 | -0.64, 0.30 | 0.24 | 0.48 | 0.18 | -0.34, 0.71 | 0.27 | 0.49 |
|  | Secondary | Ref |  |  |  | Ref |  |  |  |
|  | High school diploma | -0.04 | -0.44, 0.35 | 0.20 | 0.83 | -0.27 | -0.74, 0.19 | 0.24 | 0.25 |
|  | Higher education | -0.44 | -0.86, -0.02 | 0.21 | 0.04 | -0.62 | -1.14, -0.10 | 0.26 | 0.02 |
| Relationship status | |  |  |  |  |  |  |  |  |
|  | In a couple | Ref |  |  |  | Ref |  |  |  |
|  | Not in a couple | -0.60 | -0.90, -0.30 | 0.15 | <0.0001 | -0.45 | -0.83, -0.06 | 0.19 | 0.02 |
| Region of origin | |  |  |  |  |  |  |  |  |
|  | North Africa/Middle East |  |  |  |  | Ref |  |  |  |
|  | West Africa |  |  |  |  | -1.12 | -1.61, -0.63 | 0.25 | <0.0001 |
|  | East/Central Africa |  |  |  |  | -1.74 | -2.42, -1.07 | 0.34 | <0.0001 |
|  | Former Soviet/Yugoslavian States |  |  |  |  | -0.11 | -0.66, 0.43 | 0.28 | 0.68 |
|  | European Union |  |  |  |  | -0.21 | -0.82, 0.39 | 0.31 | 0.49 |
|  | Others |  |  |  |  | -0.40 | -1.14, 0.33 | 0.37 | 0.28 |
| Duration of residence (yrs) | |  |  |  |  |  |  |  |  |
|  | <1 | Ref |  |  |  |  |  |  |  |
|  | [1-2[ | -0.12 | -0.67, 0.43 | 0.28 | 0.66 |  |  |  |  |
|  | [2-3[ | -0.24 | -0.74, 0.26 | 0.26 | 0.34 |  |  |  |  |
|  | [3-4[ | -0.80 | -1.34, -0.26 | 0.28 | 0.004 |  |  |  |  |
|  | [4-5[ | -0.62 | -1.24, 0.00 | 0.32 | 0.05 |  |  |  |  |
|  | [5-6[ | -0.51 | -1.11, 0.08 | 0.30 | 0.09 |  |  |  |  |
|  | [6-7[ | -0.55 | -1.18, 0.08 | 0.32 | 0.09 |  |  |  |  |
|  | >= 7 | -0.95 | -1.47, -0.44 | 0.26 | <0.0001 |  |  |  |  |
| GP visit in last 12 mths | |  |  |  |  |  |  |  |  |
|  | No | Ref |  |  |  |  |  |  |  |
|  | Yes | -0.23 | -0.55-0.09 | 0.16 | 0.15 |  |  |  |  |
| Gynecologist visit in last 12 mths | |  |  |  |  |  |  |  |  |
|  | No | Ref |  |  |  |  |  |  |  |
|  | Yes | -0.51 | -0.81, -0.21 | 0.15 | 0.001 |  |  |  |  |
| Own public transportation card | |  |  |  |  |  |  |  |  |
|  | No |  |  |  |  | 0.43 | 0.07, 0.80 | 0.19 | 0.02 |
|  | Yes |  |  |  |  | Ref |  |  |  |
| Baseline odds | | 1.08 | 0.53-1.63 | 0.28 | <0.0001 | 0.11 | -0.53, 0.75 | 0.33 | 0.19 |

^1^Analyses adjusted simultaneously on all variables in the table

Rho = 0.15, p = 0.19
