# Supplementary material for: Do vulnerable groups access prevention services? Cervical cancer screening and HIV testing among homeless migrant women in the Paris metropolitan area
Source: PLoS One. 2021 Aug 13;16(8):e0255900. doi: 10.1371/journal.pone.0255900 (PMC8363022; doi:10.1371/journal.pone.0255900)
Supplement: S2 Table — (DOCX) [file pone.0255900.s002.docx]

**S2 Table.** **Characteristics associated with no lifetime cervical cancer screening and no lifetime HIV test, bivariate and multivariate logistic regression analyses, DSAFHIR study** (corresponding to Table 2)

|  |  | No lifetime cervical cancer screening | | | | | | No lifetime HIV test | | | | | |
| --- | --- | --- | --- | --- | --- | --- | --- | --- | --- | --- | --- | --- | --- |
|  |  | OR | 95% CI | aOR^1^  N=376 | 95% CI | Standard error | p-value | OR | 95% CI | aOR^1^  N=414 | 95% CI | Standard error | p-value |
| Age (yrs) | |  |  |  |  |  |  |  |  |  |  |  |  |
|  | < 30 | 1.00 |  | 1.00 |  |  |  | 1.00 |  | 1.00 |  |  |  |
|  | [30-40[ | 0.88 | 0.53-1.47 | 1.24 | 0.68-2.28 | 0.38 | 0.48 | 0.54 | 0.34-0.87 | 0.70 | 0.39-1.24 | 0.21 | 0.22 |
|  | [40-50[ | 0.50 | 0.26-1.47 | 0.86 | 0.38-1.92 | 0.35 | 0.71 | 0.95 | 0.53-1.73 | 1.22 | 0.58-2.57 | 0.46 | 0.60 |
|  | 50 + | 1.08 | 0.44-2.65 | 3.18 | 0.98-10.3 | 1.91 | 0.05 | 1.12 | 0.47-2.67 | 0.72 | 0.22-2.36 | 0.44 | 0.59 |
| Education | |  |  |  |  |  |  |  |  |  |  |  |  |
|  | No schooling | 2.37 | 1.20-4.69 | 1.94 | 0.87-4.33 | 0.79 | 0.11 | 1.27 | 0.66-2.44 | 1.72 | 0.78-3.81 | 0.70 | 0.17 |
|  | Primary | 0.86 | 0.44-1,67 | 0.74 | 0.35-1.57 | 0.28 | 0.43 | 1.09 | 0.56-2.10 | 1.38 | 0.63-3.01 | 0.55 | 0.42 |
|  | Secondary | 1.00 |  | 1.00 |  |  |  | 1.00 |  | 1.00 |  |  |  |
|  | High school diploma | 0.82 | 0.46-1.44 | 0.84 | 0.44-1.60 | 0.28 | 0.59 | 0.60 | 0.34-1.07 | 0.66 | 0.33-1.31 | 0.23 | 0.23 |
|  | Higher education | 0.46 | 0.25-0.86 | 0.46 | 0.23-0.93 | 0.16 | 0.03 | 0.45 | 0.23-0.87 | 0.43 | 0.20-0.95 | 0.17 | 0.03 |
| Relationship status | |  |  |  |  |  |  |  |  |  |  |  |  |
|  | In a couple | 1.00 |  | 1.00 |  |  |  | 1.00 |  | 1.00 |  |  |  |
|  | Not in a couple | 0.52 | 0.35-0.79 | 0.46 | 0.26-0.81 | 0.13 | 0.007 | 0.27 | 0.17-0.43 | 0.57 | 0.32-1.03 | 0.17 | 0.06 |
| Region of origin | |  |  |  |  |  |  |  |  |  |  |  |  |
|  | North Africa/Middle East | 1.00 |  | 1.00 |  |  |  | 1.00 |  | 1.00 |  |  |  |
|  | West Africa | 1.08 | 0.58-2.00 | 1.24 | 0.57-2.68 | 0.49 | 0.59 | 0.23 | 0.12-0.44 | 0.15 | 0.07-0.33 | 0.06 | <0.0001 |
|  | East/Central Africa | 0.53 | 0.27-1.06 | 0.78 | 0.33-1.83 | 0.34 | 0.57 | 0.05 | 0.02-0.13 | 0.06 | 0.02-0.20 | 0.04 | <0.0001 |
|  | Former Soviet/Yugoslavian States | 0.59 | 0.27-1.29 | 0.48 | 0.20-1.17 | 0.22 | 0.11 | 0.79 | 0.36-1.73 | 0.91 | 0.40-2.07 | 0.38 | 0.81 |
|  | European Union | 2.71 | 1.13-6.50 | 2.62 | 0.94-7.27 | 1.36 | 0.06 | 1.47 | 0.67-3.25 | 0.85 | 0.36-2.02 | 0.38 | 0.71 |
|  | Others | 1.87 | 0.67-5.18 | 2.02 | 0.62-6.55 | 1.21 | 0.24 | 0.61 | 0.24-1.57 | 0.71 | 0.26-1.96 | 0.37 | 0.51 |
| Duration of residence (yrs) | |  |  |  |  |  |  |  |  |  |  |  |  |
|  | <1 | 1.00 |  | 1.00 |  |  |  | 1.00 |  |  |  |  |  |
|  | [1-2[ | 0.50 | 0.23-1.09 | 0.55 | 0.22-1.35 | 0.25 | 0.19 | 0.63 | 0.29-1.38 |  |  |  |  |
|  | [2-3[ | 0.51 | 0.24-1.08 | 0.54 | 0.23-1.24 | 0.23 | 0.15 | 0.63 | 0.30-1.33 |  |  |  |  |
|  | [3-4[ | 0.20 | 0.09-0.44 | 0.21 | 0.09-0.53 | 0.10 | 0.001 | 0.79 | 0.37-1.69 |  |  |  |  |
|  | [4-5[ | 0.32 | 0.13-0.78 | 0.32 | 0.11-0.89 | 0.17 | 0.02 | 0.65 | 0.28-1.53 |  |  |  |  |
|  | [5-6[ | 0.33 | 0.14-0.79 | 0.31 | 0.11-0.82 | 0.15 | 0.01 | 0.80 | 0.34-1.86 |  |  |  |  |
|  | [6-7[ | 0.48 | 0.19-1.19 | 0.46 | 0.16-1.28 | 0.24 | 0.13 | 0.85 | 0.34-2.12 |  |  |  |  |
|  | >= 7 | 0.23 | 0.12-0.47 | 0.17 | 0.07-0.39 | 0.07 | <0.0001 | 1.35 | 0.71-2.55 |  |  |  |  |
| GP visit in last 12 mths | |  |  |  |  |  |  |  |  |  |  |  |  |
|  | No | 1.00 |  | 1.00 |  |  |  |  |  |  |  |  |  |
|  | Yes | 0.59 | 0.39-0.91 | 0.65 | 0.39-1.08 | 0.17 | 0.09 |  |  |  |  |  |  |
| Gynecologist visit in last 12 mths | |  |  |  |  |  |  |  |  |  |  |  |  |
|  | No | 1.00 |  | 1.00 |  |  |  | 1.00 |  |  |  |  |  |
|  | Yes | 0.45 | 0.30-0.68 | 0.49 | 0.30-0.80 | 0.12 | 0.004 | 0.73 | 0.48-1.11 |  |  |  |  |
| Own public transportation card | |  |  |  |  |  |  |  |  |  |  |  |  |
|  | No | 1.36 | 0.89-2.08 |  |  |  |  | 1.84 | 1.17-2.89 | 1.72 | 1.00-2.98 | 0.48 | 0.05 |
|  | Yes | 1.00 |  |  |  |  |  | 1.00 |  | 1.00 |  |  |  |
| Baseline odds | |  |  | 5.13 | 1.85-14.2 | 2.67 | 0.002 |  |  | 1.40 | 0.55-3.55 | 0.66 | 0.47 |
