# Supplementary material for: Do vulnerable groups access prevention services? Cervical cancer screening and HIV testing among homeless migrant women in the Paris metropolitan area
Source: PLoS One. 2021 Aug 13;16(8):e0255900. doi: 10.1371/journal.pone.0255900 (PMC8363022; doi:10.1371/journal.pone.0255900)
Supplement: S3 Table — (DOCX) [file pone.0255900.s003.docx]

**S3 Table.** **Mean time since last HIV test (in years), multivariate linear regression, by region of origin, DSAFHIR study** (corresponding to table 3)

|  |  | All  n = 268 | | | | Sub Saharan  n = 200 | | | | Non-Sub Saharan  n = 68 | | | |
| --- | --- | --- | --- | --- | --- | --- | --- | --- | --- | --- | --- | --- | --- |
|  |  | Coef | 95% CI | Standard error | p | Coef | 95% CI | Standard error | p | Coef | 95% CI | Standard error | p |
| Duration of residence (yrs) | | 0.21 | 0.09, 0.33 | 0.05 | 0.001 | 0.19 | 0.04, 0.34 | 0.08 | 0.01 | 0.27 | 0.07, 0.47 | 0.10 | 0.01 |
| Educational level | | -0.04 | -0.26, 0.16 | 0.11 | 0.64 | -0.12 | -0.36, 0.13 | 0.12 | 0.35 | 0.10 | -0.25, 0.45 | 0.17 | 0.57 |
| Age | | 0.42 | -0.002, 0.84 | 0.21 | 0.05 | 0.50 | -0.08, 1.09 | 0.30 | 0.09 | 0.41 | -0.24, 1.05 | 0.32 | 0.21 |
| Relationship status | | 0.66 | 0.20, 1.12 | 0.23 | 0.005 | 0.37 | -0.16, 0.89 | 0.26 | 0.16 | 1.68 | 0.48, 2.87 | 0.60 | 0.007 |
| GP visit in last 12 mths | | -0.11 | -0.66, 0.44 | 0.28 | 0.70 | 0.07 | -0.57, 0.70 | 0.32 | 0.83 | -0.72 | -1.98, 0.54 | 0.63 | 0.26 |
| Gynecologist visit in last 12 mths | | -1.16 | -1.62, -0.71 | 0.23 | <0.0001 | -1.12 | -1.67, -0.56 | 0.28 | <0.0001 | -1.45 | -2.32, -0.58 | 0.43 | 0.001 |
| Baseline odds | | 0.68 | -0.22, 1.58 | 0.46 | 0.13 | 0.79 | -0.34, 1.91 | 0.57 | 0.17 | -0.21 | -1.91, 1.50 | 0.85 | 0.80 |
